# Structure of the outer membrane porin OmpW from the pervasive pathogen *Klebsiella pneumoniae*

**DOI:** 10.1107/S2053230X23010579

**Published:** 2024-01-01

**Authors:** Chloe Seddon, Gad Frankel, Konstantinos Beis

**Affiliations:** a Department of Life Sciences, Imperial College, London, United Kingdom; b Rutherford Appleton Laboratory, Research Complex at Harwell, Didcot OX11 0FA, United Kingdom; University of Toronto, Canada

**Keywords:** *Klebsiella pneumoniae*, outer membrane porin, OmpW, bacterial conjugation, β-barrel

## Abstract

Conjugation is the process by which plasmids, including those that carry antibiotic-resistance genes, are mobilized from one bacterium (the donor) to another (the recipient). The conjugation efficiency of IncF-like plasmids relies on the formation of mating-pair stabilization via intimate interactions between outer membrane proteins on the donor (a plasmid-encoded TraN isoform) and recipient bacteria. Conjugation of the R100-1 plasmid into *Escherichia coli* and *Klebsiella pneumoniae* (KP) recipients relies on pairing between the plasmid-encoded TraNα in the donor and OmpW in the recipient. Here, the crystal structure of *K. pneumoniae* OmpW (OmpW_KP_) is reported at 3.2 Å resolution. OmpW_KP_ forms an eight-stranded β-barrel flanked by extracellular loops. The structures of *E. coli* OmpW (OmpW_EC_) and OmpW_KP_ show high conservation despite sequence variability in the extracellular loops.

## Abstract 2

The crystal structure of the outer membrane protein OmpW from *Klebsiella pneumoniae* is reported.

## 1. Introduction

Outer membrane porins (OMPs) are an important class of β-barrel proteins that form water-filled channels in Gram-negative bacteria. They enable the diffusion of nutrients and the efflux of toxins across the outer membrane (Lou *et al.*, 2009 ▸). From a clinical perspective, OMPs are important in modulating the diffusion of antibiotics into the bacterial cell, where mutations or reduced expression of the OMPs enhance antibiotic resistance (Pagès *et al.*, 2008 ▸). It has also been shown that OMPs participate in F-like plasmid conjugation, a form of horizontal gene transfer where plasmids are transferred from donor to recipient bacteria in a contact-dependent manner (Lederberg & Tatum, 1946 ▸; Frankel *et al.*, 2023 ▸). We have recently shown that the efficient conjugation of the multidrug-resistant R100-1 plasmid into both *Escherichia coli* (EC) and *Klebsiella pneumoniae* (KP) relies on the formation of mating-pair stabilization via interaction between the R100-1-encoded OM protein TraNα in the donor and the OMP OmpW_EC_ or OmpW_KP_ in the recipient (Low *et al.*, 2022 ▸, 2023 ▸). Pairing of the TraN isoform with recipient receptors mediates conjugation species specificity and host range; an in-depth review of mating-pair stabilization and the role of TraN has been provided by Frankel *et al.* (2023 ▸). In brief, TraN is an outer membrane protein that is composed of two domains, a base and an extended tip; the base consists of a conserved amphipathic α-helix that possibly anchors TraN to the OM, whereas the tip is mostly comprised of β-sheets linked to a β-sandwich domain. The loops at the tip function as a TraN sensor that participates in recipient selection (Frankel *et al.*, 2023 ▸)

In addition to its role in conjugation, OmpW contributes to virulence as the upregulation of OmpW_EC_ increases resistance to host immune defence (Wu *et al.*, 2013 ▸). Conversely, OmpW is a key antigen; indeed, OmpW-immunized mice show greater protection against bacterial infections. This could pave the way for the use of OmpW in vaccine preparation (Huang *et al.*, 2015 ▸).

The crystal structure of OmpW_EC_ forms an eight-stranded monomeric β-barrel with an extracellular region that is involved in hydrophobic substrate binding (Hong *et al.*, 2006 ▸). Here, we present the crystal structure of OmpW_KP_ at 3.2 Å resolution and draw structural comparisons with OmpW_EC_, both of which are conjugation receptors for TraNα.

## 2. Materials and methods

### 2.1. Macromolecule production

The mature protein sequence of OmpW_KP_ (His22–Phe212) was subcloned into the pTAMANHISTEV vector in-frame with a *tamA* signal sequence followed by an N-terminal His_7_ tag and a Tobacco etch virus (TEV) cleavage site, using the NcoI and XhoI restriction-enzyme sites. The construct was transformed into *E. coli* BL21 C43(DE3) competent cells [F^−^ *ompT hsd_SB_* 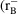 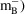 *gal dcm* (DE3)] (Miroux & Walker, 1996 ▸) and expressed in Terrific Broth (TB) medium. Cultures were incubated at 37°C with orbital shaking at 200 rev min^−1^ until an optical density at 600 nm (OD_600_) of 0.6–0.8 was achieved. Cultures were then induced with isopropyl β-d-1-thiogalactopyranoside (IPTG) at a final concentration of 1 m*M* and maintained for 3 h. The cells were harvested by centrifugation (Beckman Coulter) at 8000*g* for 10 min and stored at −80°C. Outer membranes were prepared as described previously (Beis *et al.*, 2006 ▸) and were then solubilized in phosphate-buffered saline (PBS) supplemented with 1% *N*,*N*-dimethyl-*n*-dodecylamine *N*-oxide (LDAO) overnight. Unsolubilized membranes and debris were removed by ultracentrifugation at 131 000*g* for 1 h. The supernatant was supplemented with 30 m*M* imidazole and passed through a 5 ml HisTrap HP column (Cytiva) equilibrated in PBS with 0.1% LDAO. The column was washed with 20 column volumes of buffer consisting of PBS, 300 m*M* NaCl, 30 m*M* imidazole pH 7.0 and 0.45% 1-*O*-(*n*-octyl)-tetraethyleneglycol (C_8_E_4_) to exchange the detergent. OmpW_KP_ was eluted in buffer consisting of 250 m*M* imidazole and 0.45% C_8_E_4_. OmpW_KP_ was then exchanged into 50 m*M* NaCl, 10 m*M* HEPES pH 7.0 and 0.45% C_8_E_4_ using a PD-10 Desalting Column (Cytiva) and concentrated to 15 mg ml^−1^. Macromolecule-production information is summarized in Table 1 ▸.

### 2.2. Crystallization

Purified OmpW_KP_ underwent preliminary screening by the sitting-drop vapour-diffusion method at 293 K using the sparse-matrix MemGold screen (Molecular Dimensions). The protein was mixed with the precipitant in a 1:1 ratio using a Mosquito LCP crystallization robot (SPT Labtech). Orthorhombic crystals appeared after 24 h in the following condition: 0.35 *M* lithium sulfate, 0.1 *M* sodium acetate pH 4.0, 11% PEG 600. Large OmpW_KP_ crystals were obtained by the hanging-drop vapour-diffusion method. Crystals were cryoprotected in a mixture of well solution supplemented with 30% PEG 600.

### 2.3. Data collection and processing

Diffraction data were collected on the I03 beamline at Diamond Light Source (DLS), Didcot, United Kingdom using an EIGER2 XE 16M detector. The crystals belonged to space group *C*222. Diffraction frames were indexed and integrated using the *DIALS* pipeline as implemented at DLS (Winter *et al.*, 2018 ▸). The data were scaled using *AIMLESS* in the *CCP*4 suite (Evans & Murshudov, 2013 ▸; Agirre *et al.*, 2023 ▸). The data-collection parameters and merging statistics are summarized in Table 2 ▸.

### 2.4. Structure solution, model building and refinement

The structure of OmpW_KP_ was solved by molecular replace­ment with the *AlphaFold*-predicted model of OmpW_KP_ (Jumper *et al.*, 2021 ▸) using *Phenix* (Liebschner *et al.*, 2019 ▸). The calculated Matthews coefficient (*V*_M_) was 3.84 Å^3^ Da^−1^, suggesting the presence of one molecule of OmpW_KP_ in the asymmetric unit; this corresponds to a solvent content of 68% by volume. Manual adjustments to the model were performed in *Coot* (Emsley *et al.*, 2010 ▸). Density for two sulfate ions was present and they were included in the model. *Phenix* was used for refinement (Afonine *et al.*, 2018 ▸). *MolProbity* was used for validation (Williams *et al.*, 2018 ▸). Figure preparation was performed using *UCSF ChimeraX* 1.6 (Pettersen *et al.*, 2021 ▸). Refinement statistics are summarized in Table 3 ▸.

## 3. Results and discussion

### 3.1. Purification and crystallization of OmpW_KP_

**Figure 1:**
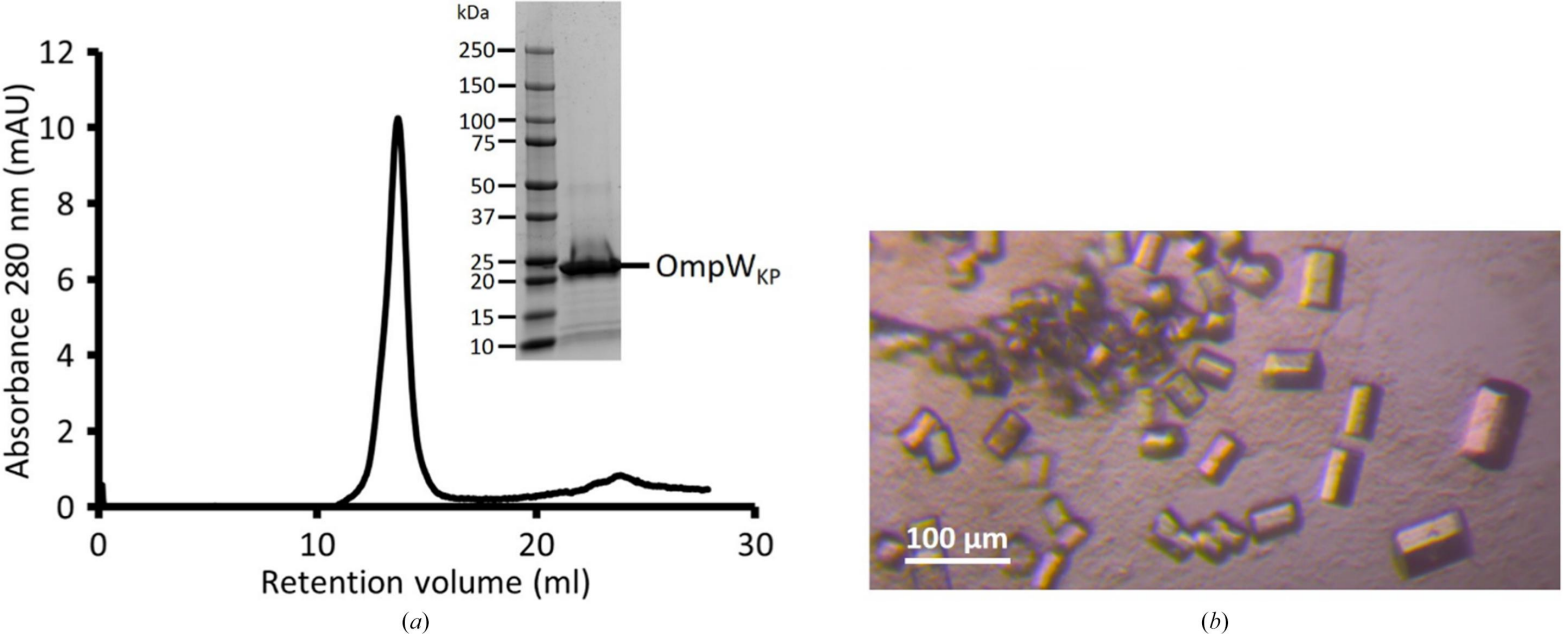
Purification and crystallization of OmpW_KP_. (*a*) SEC analysis of OmpW_KP_ shows a monodisperse peak, with SDS–PAGE analysis of purified OmpW_KP_; the purity is greater than 95%. (*b*) Orthorhombic OmpW_KP_ crystals. The largest crystals had dimensions of 100 × 20 × 20 µm.

OmpW_KP_ was overexpressed in *E. coli* and purified in C_8_E_4_ to homogeneity by immobilized metal affinity chromatography. OmpW_KP_ displays a monodisperse peak on size-exclusion chromatography and was >95% pure as judged by SDS–PAGE (Fig. 1 ▸ *a*). OmpW_KP_ crystals grew overnight from a solution consisting of 0.35 *M* lithium sulfate, 0.1 *M* sodium acetate pH 4.0, 11%(*w*/*v*) PEG 600 (Fig. 1 ▸ *b*). The crystals had an orthorhombic shape and were further optimized by the hanging-drop vapour-diffusion method. The optimized crystals diffracted X-rays to 3.2 Å resolution and belonged to space group *C*222.

### 3.2. Structure solution of OmpW_KP_

**Figure 2:**
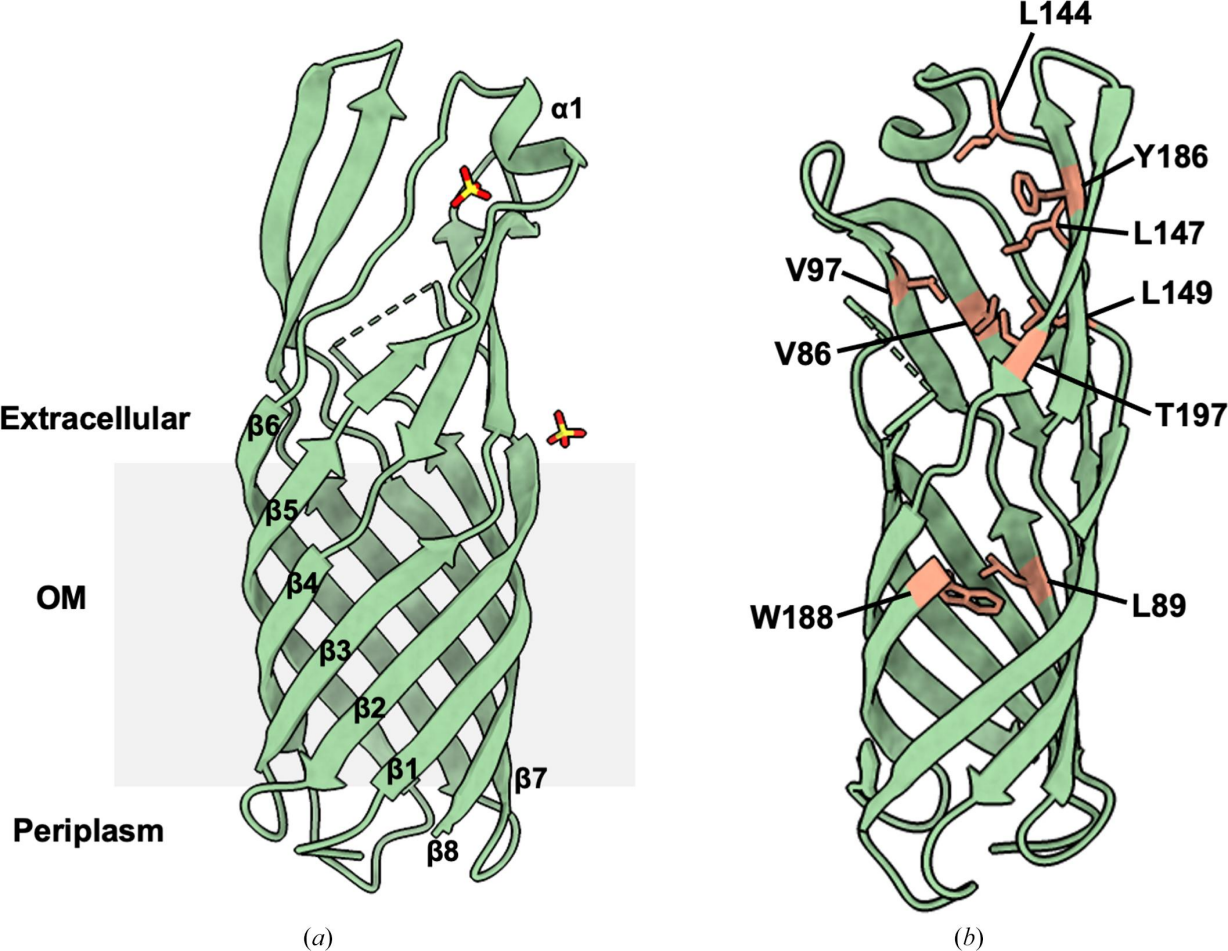
Structure of OmpW_KP_. (*a*) Cartoon representation of the OmpW_KP_ structure (shown in green) perpendicular to the OM (depicted in grey). Sulfate ions are depicted as sticks (O atoms are shown in red and S atoms in yellow). The missing residues are marked with a green dashed line. (*b*) The hydrophobic residues lining the extracellular region and forming the hydrophobic gate, Leu89 and Trp188, are shown as orange sticks.

The structure of OmpW_KP_ was solved by molecular replace­ment using the *AlphaFold*-predicted model. Continuous electron density could be observed for most of the structure except for Gly41–Phe52, which were omitted from model building. The OmpW_KP_ structure consists of eight antiparallel β-strands (β1–β8) that arrange to form a hollow β-barrel in the OM and an extracellular solvent-exposed region (Fig. 2 ▸ *a*). The extracellular region is formed from the extended β-strands of the barrel and a single α-helical turn (α1) connecting β5 and β6. A hydrophobic gate is present midway through the channel consisting of residues Leu89 and Trp188, as in OmpW_EC_ (Hong *et al.*, 2006 ▸), where the extracellular entrance to the channel is lined with hydrophobic residues (Fig. 2 ▸ *b*).

### 3.3. Comparison of OmpW_KP_ with OmpW_EC_

**Figure 3:**
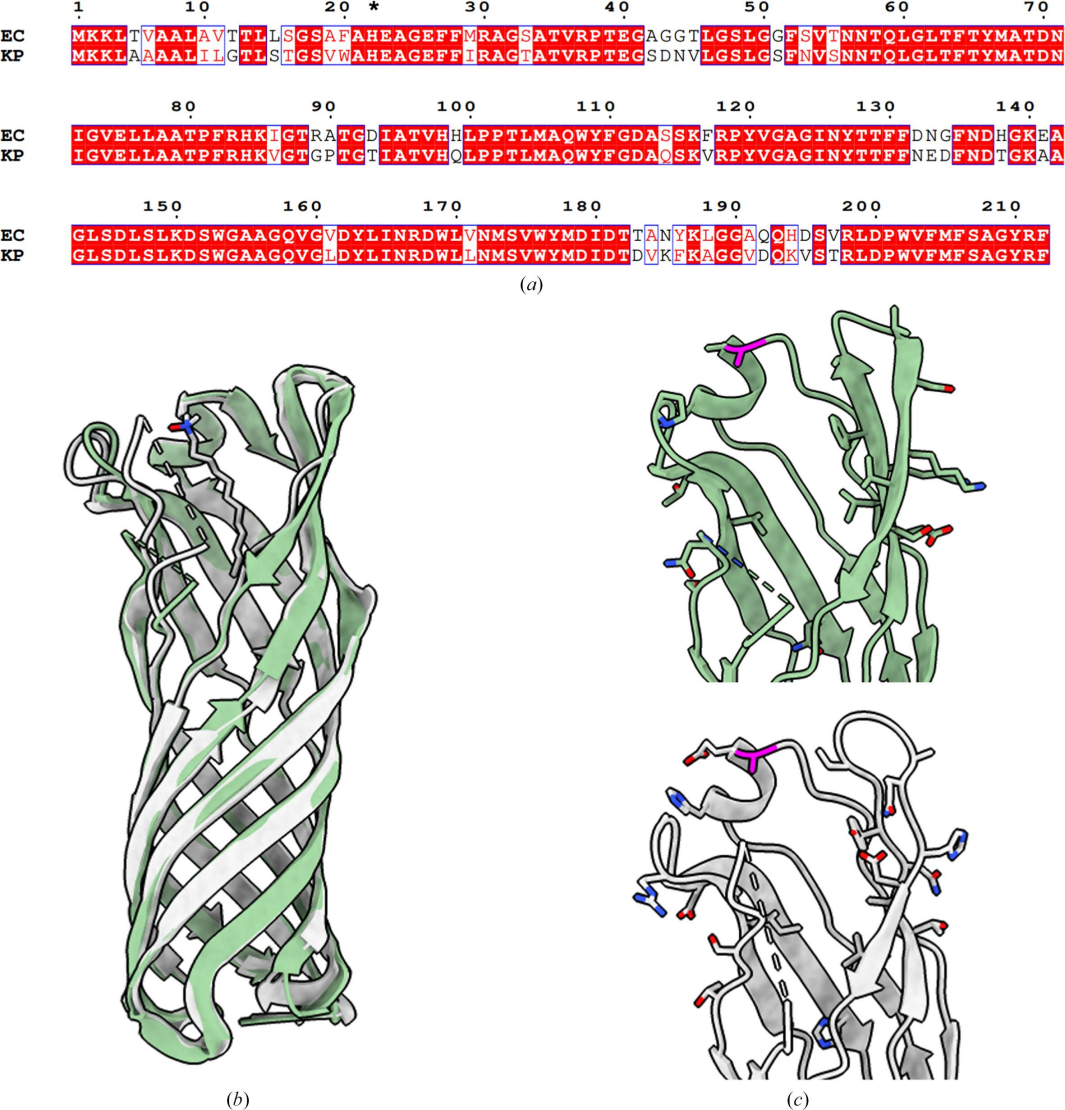
Sequence alignment and superimposition of OmpW_KP_ with OmpW_EC_. (*a*) A sequence alignment of OmpW_EC_ (UniProt ID P0A915) and OmpW_KP_ (UniProt ID W9B759) is shown; conserved and similar residues are shown in red and blue boxes, respectively. Residue numbers are indicated above the protein sequences. An asterisk indicates the mature protein after cleavage of the signal peptide. The alignment was prepared using *ESPript* (Robert & Gouet, 2014 ▸). (*b*) OmpW_KP_ (green) superimposed with OmpW_EC_ (grey; PDB entry 2f1v; Hong *et al.*, 2006 ▸) shows high structural conservation. The LDAO molecule bound to OmpW_EC_ is shown as sticks. (*c*) Close-up view of the extracellular regions of OmpW_KP_ (green) and OmpW_EC_ (grey), with the side chains of amino-acid differences shown as stick models. The conserved Ala142 is shown in magenta.

The closest structural homologue to OmpW_KP_ is OmpW_EC_, which shares 82.7% sequence identity and 88% sequence similarity (Fig. 3 ▸ *a*). The two structures can be superimposed with an r.m.s.d. of 0.54 Å over 171 C^α^ atoms (Fig. 3 ▸ *b*); they show high structural conservation of the β-barrel, with minor differences confined to the extracellular region, which displays some flexibility. The extracellular loop 1 that connects β1 and β2 is missing in both the OmpW_KP_ and the OmpW_EC_ structures, suggesting a highly flexible structure. This flexibility could be associated with substrate recruitment, as the conformation of the modelled loop 1 blocks the channel in the *AlphaFold*-predicted structure. In the OmpW_EC_ structure an LDAO molecule is bound at the extracellular region but loop 1 is not fully resolved, suggesting that the inherited flexibility cannot be stabilized upon its binding (Hong *et al.*, 2006 ▸). This highly mobile structural element on the extracellular loop is likely to shield the hydrophobic face of the extracellular region and it could transiently open to recruit hydrophobic substrates. Despite the sequence conservation of loop 1 being low between OmpW_KP_ and OmpW_EC_, this suggests that it might be involved in substrate selectivity between different bacterial species.

Despite amino-acid differences in the extracellular region between OmpW_KP_ and OmpW_EC_ (Fig. 3 ▸ *c*), where the tip of TraN_R100-1_ has been shown to bind (Low *et al.*, 2023 ▸), binding of TraN_R100-1_ is not impaired between the two species. We previously reported that Ala142, which is conserved between OmpW_KP_ and OmpW_EC_, acts as the minimum residue for specificity towards TraN_R100-1_ (Low *et al.*, 2023 ▸); the equivalent residue in *Citrobacter rodentium* OmpW (OmpW_CR_) is Asn142, which prevents R100-1 conjugation because of a steric clash with the tip of TraN_R100-1_ (Low *et al.*, 2023 ▸). The N142A mutation in OmpW_CR_ restored conjugation efficiency (Low *et al.*, 2023 ▸).

In conclusion, we have resolved the crystal structure of OmpW_KP_; structural comparison with OmpW_EC_ identified the presence of a highly flexible loop, loop 1, that might be important for shielding the pore prior to hydrophobic substrate recruitment. In addition, despite sequence and structural differences in the extracellular region, both porins can mediate interactions with TraNα.

## Supplementary Material

PDB reference: OmpW from *Klebsiella pneumoniae*, 8qxp

## Figures and Tables

**Table 1:** OmpW_KP_ construct design

Source organism	*Klebsiella pneumoniae*
DNA source	*K. pneumoniae* ICC8001
Forward primer†	CATGCCATGGGTCATGAGGCGGGGGAGTTTTTC
Reverse primer‡	CCGCTCGAGTTAGAACCGATAGCCTGCGGAGAA
Cloning vector	pTAMANHISTEV
Expression vector	pTAMANHISTEV
Expression host	*E. coli*
Complete amino-acid sequence of the construct produced§	MRYIRQLCCVSLLCLSGSAAAANVRLQHHHHHHHDYDIPTTENLYFQGAMGHEAGEFFIRAGTATVRPTEGSDNVLGSLGSFNVSNNTQLGLTFTYMATDNIGVELLAATPFRHKVGTGPTGTIATVHQLPPTLMAQWYFGDAQSKVRPYVGAGINYTTFFNEDFNDTGKAAGLSDLSLKDSWGAAGQVGLDYLINRDWLLNMSVWYMDIDTDVKFKAGGVDQKVSTRLDPWVFMFSAGYRF

† The NcoI restriction site is underlined.

‡ The XhoI restriction site is underlined.

§ The pTAMA signal sequence that is not present after cleavage is underlined.

**Table 2:** Data collection and processing Values in parentheses are for the outer shell.

Diffraction source	I03, DLS
Wavelength (Å)	0.9763
Temperature (K)	100
Detector	EIGER2 XE 16M
Space group	*C*222
*a*, *b*, *c* (Å)	87.92, 138.63, 52.96
α, β, γ (°)	90.0, 90.0, 90.0
Mosaicity (°)	0.15
Resolution range (Å)	52.9–3.2 (3.3–3.2)
Total No. of reflections	71778 (7496)
No. of unique reflections	5639 (560)
Completeness (%)	100 (100)
Multiplicity	12.7 (13.4)
CC_1/2_	0.85 (0.99)
〈*I*/σ(*I*)〉	64 (2.5)
*R*_r.i.m._	0.082 (0.207)
Overall *B* factor from Wilson plot (Å^2^)	78.7

**Table 3:** Structure solution and refinement Values in parentheses are for the outer shell.

Resolution range (Å)	52.97–3.20 (3.31–3.20)
Completeness (%)	100 (100)
No. of reflections, working set	5633 (559)
No. of reflections, test set	236 (25)
Final *R*_cryst_	0.2668 (0.2646)
Final *R*_free_	0.3117 (0.3636)
No. of non-H atoms	
Protein	1388
Ion	10
Total	1398
R.m.s. deviations	
Bond lengths (Å)	0.003
Angles (°)	0.622
Average *B* factors (Å^2^)	77.7
Protein	77.5
Ion	101.8
Ramachandran plot	
Most favoured (%)	95.98
Allowed (%)	3.45
Outliers (%)	0.57 [Pro113]
